# Processing of projections containing phase contrast in laboratory micro-computerized tomography imaging

**DOI:** 10.1107/S002188981300558X

**Published:** 2013-06-07

**Authors:** Zdenko Zápražný, Dušan Korytár, Petr Mikulík, Vladimír Áč

**Affiliations:** a Institute of Electrical Engineering, Slovak Academy of Sciences, Dúbravská cesta 9, SK-841 04 Bratislava, Slovakia; b Central European Institute of Technology (CEITEC), Masaryk University, Kotlářská 2, CZ-61137 Brno, Czech Republic; c Alexander Dubček University of Trenčín, Študentská 2, SK-911 50 Trenčín, Slovakia

**Keywords:** phase-contrast imaging, X-ray imaging, X-ray radiography, digital radiography, computerized tomography, computed radiography

## Abstract

Free-space-propagation-based imaging belongs to several techniques for achieving phase contrast in the hard X-ray range. The basic precondition is to use an X-ray beam with a high degree of coherence. Although the best sources of coherent X-rays are synchrotrons, spatially coherent X-rays emitted from a sufficiently small spot of laboratory microfocus or sub-microfocus sources allow the transfer of some of the modern imaging techniques from synchrotrons to laboratories. Spatially coherent X-rays traverse a sample leading to a phase shift. Beam deflection induced by the local change of refractive index may be expressed as a dark–bright contrast on the edges of the object in an X-ray projection. This phenomenon of edge enhancement leads to an increase in spatial resolution of X-ray projections but may also lead to unpleasant artefacts in computerized tomography unless phase and absorption contributions are separated. The possibilities of processing X-ray images of lightweight objects containing phase contrast using phase-retrieval methods in laboratory conditions are tested and the results obtained are presented. For this purpose, simulated and recorded X-ray projections taken from a laboratory imaging system with a microfocus X-ray source and a high-resolution CCD camera were processed and a qualitative comparison of results was made.

## Abstract 2

Processing of phase-contrast images in laboratory conditions is described.

## 1. Introduction

Modern laboratory X-ray imaging systems with a microfocus source and a CCD camera make it possible to move some of the modern imaging techniques from synchrotrons to laboratories (Gundogdu *et al.*, 2007 ▶). The main stream of our work is to study and to implement advanced phase-contrast imaging techniques in laboratory conditions. For this purpose, it was necessary to design and to build a convenient X-ray imaging system (Zaprazny *et al.*, 2012 ▶). This system is suitable for lightweight objects. Such objects can be biological objects, plastics, wood, paper *etc.*, where the phase contrast helps increase the visibility of the finest structures. Phase contrast is simply achieved by propagation of spatially coherent X-rays through free space (Wilkins *et. al.*, 1996 ▶). The most common designation of this technique is propagation-based phase-contrast imaging (Gureyev *et al.*, 2009 ▶). Phase-contrast images were obtained using this technique and were applied as the input in an algorithm for recovery of the phase information.

## 2. Experimental setup

The design of the X-ray imaging system was based on the key requirements for high-resolution X-ray computerized tomography (CT). The requirements are as follows: a high-resolution X-ray detector, a high-resolution goniometer and a small spot size of the X-ray source. The use of a Newport high-resolution rotation stage (with minimal incremental motion of 0.0002°) for the sample holder allows sequential CT projections to be taken. The CCD X-ray mini FDI camera (Photonic Science) was used as a detector. The basic parameters of the camera are as follows: number of pixels: 1392 × 1040; input pixel size: 6.4 × 6.4 µm; active area: 10 × 8 mm; Gd_2_O_2_S:Tb_3_ scintillator of 15 µm thickness with optimal energy response in the range 5–17 keV. A focus size of 8 µm is declared for the transmission tungsten anode of the X-ray source. The source emits a conical X-ray beam of space angle 39°, in a voltage range up to 80 kV and a current range up to 100 µA. The X-ray imaging system allows a magnification factor in the range of 1.1–140 to be obtained, corresponding to an effective pixel size in the range 5.8–0.05 µm. These values do not match the spatial resolution of the X-ray imaging system owing to a blur aberration of the X-ray beam. The real spatial resolution of the X-ray imaging system is down to 3 µm (Zápražný *et al.*, 2011 ▶). The lateral coherence lengths are in the range of 0.3–13.5 µm in the case of a polychromatic X-ray spectrum. This property of X-rays allows the visibility (contrast) of some features of the objects to be increased by means of edge visibility enhancement (phase contrast).

### 2.1. Software

The software tools *SRCLsim* (Helfen *et al.*, 2005 ▶, 2011 ▶), *X-TRACT* (http://www.ts-imaging.net/Services/AppInfo/X-TRACT.aspx), *Octopus* (Vlassenbroeck *et al.*, 2007 ▶) and *VGStudio* (http://www.volumegraphics.com/en/products/vgstudio-max.html) for simulations and for processing of X-ray projections were used.

*SRCLsim* is a forward-imaging simulation program for CT, laminography and tomosynthesis (CT with an inclined axis). The image calculated can be a pure transmission image or a phase-contrast image.

*X-TRACT* is an image analysis and processing application (Gureyev *et al.*, 2011 ▶). It contains tools for pre-processing, phase retrieval and tomographic reconstruction. This software was primarily used for recovery of the optical phase of an X-ray beam from a one intensity image. It currently implements more than 20 algorithms for phase and/or amplitude extraction, *e.g.* transport-of-intensity-based algorithms, Born- and Rytov-based Fourier optics type methods, and Gerchberg–Saxton–Fienup type algorithms.

The *Octopus* software is a reconstruction package for the processing of tomography data acquired in almost any geometry (parallel beam, fan beam, cone beam, helical cone beam, laminography). The package was used mainly for processing of X-ray projections to CT slices. It allows a tuning of the reconstruction parameters by evaluating a single CT slice before processing the complete volume.

*VGStudio* 2.1.5 software was used for stacking of CT slices and for final three-dimensional rendering and visualization. The output of this package is a set of voxel data, providing the additional possibility of qualitative evaluation of the X-ray imaging process and of creating animations. There are even very useful measurement features available for distances, angles *etc.*

## 3. Simulations

**Figure 1:**
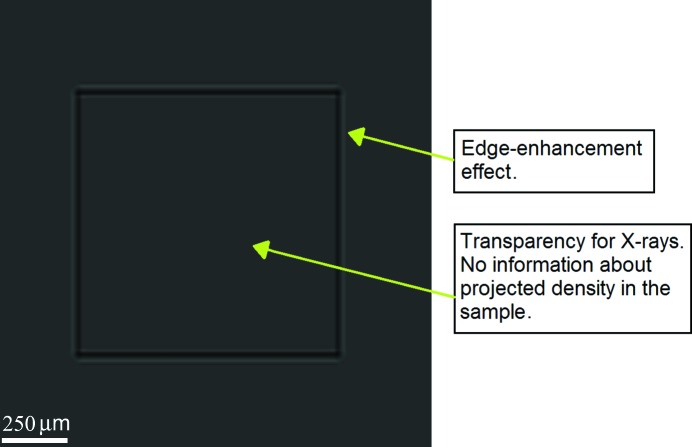
Single projection of 30 µm Kapton foil showing very poor contrast. The simulation settings were SDD = 109.3 mm and SOD = 28 mm, with the energy spectrum 5–17 keV.

The phase-contrast X-ray imaging techniques include radiography (two-dimensional) and tomography (three-dimensional). The keynote expression of phase contrast in a two-dimensional projection is the edge-enhancement effect. A simulation of this effect is shown in Fig. 1 ▶. A Kapton foil sample was used as the phase-contrast object. The sample is identifiable only because of the edge-enhancement effect without considering absorption. The sample X-ray absorption is low and it gives practically no relevant information about the projected density inside the sample. Samples made of polymethylmethacrylate (PMMA) and Kapton were chosen for simulations, because the results are appropriate for comparison with real experimental results.

**Figure 2:**
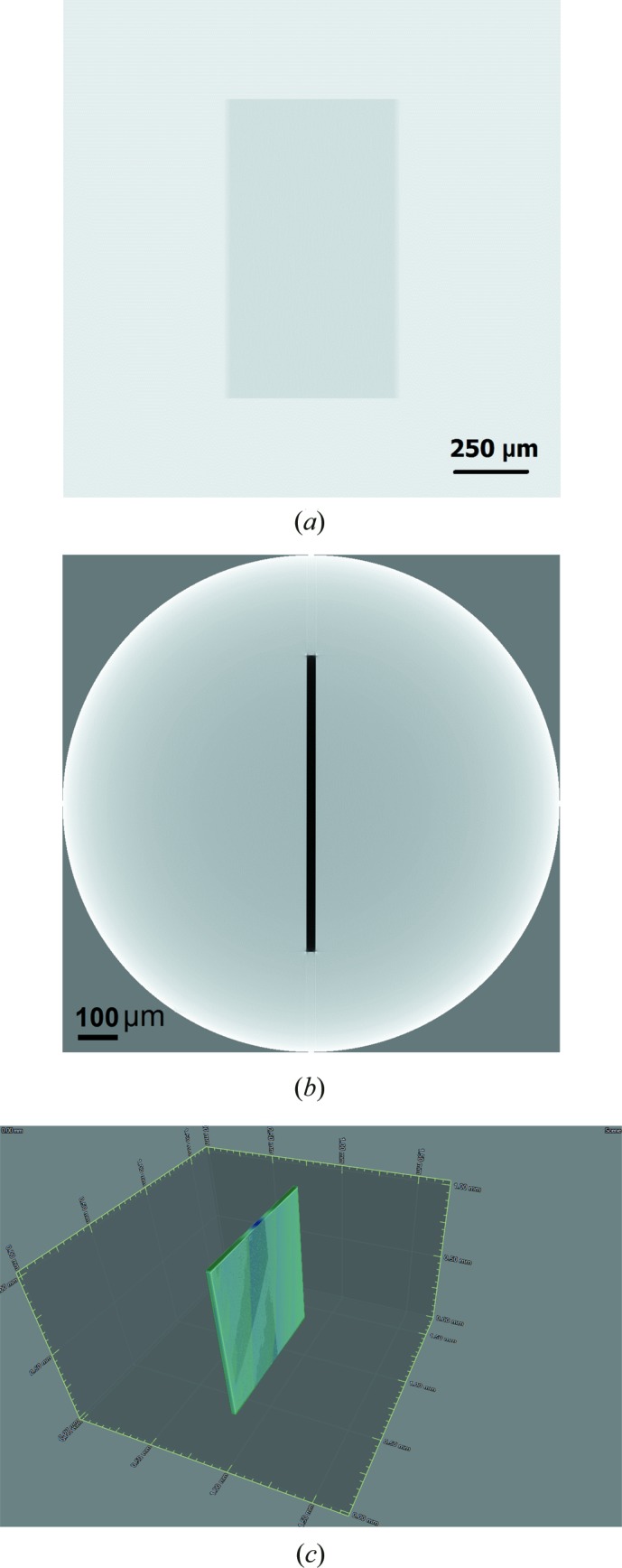
(*a*) Simulated X-ray projections of a sample made of polyimide C_22_H_10_N_2_O_5_ (Kapton foil, thickness of 30 µm) using *SRCLsim*. SDD = 109.3 mm, SOD = 28 mm and energy spectrum 5–17 keV. Total number of projections 2250. (*b*) Reconstructed slice obtained using *Octopus*. (*c*) Segmentation using *VGStudio*.

The next simulation of X-ray projections was made taking account of the absorption phenomenon to come close to the real experiment. A single X-ray simulated projection of Kapton foil in absorption mode is shown in Fig. 2 ▶(*a*). Fig. 2 ▶(*b*) shows the reconstructed CT slice using the *Octopus* software and Fig. 2 ▶(*c*) shows a visualization using the *VGStudio* tool.

**Figure 3:**
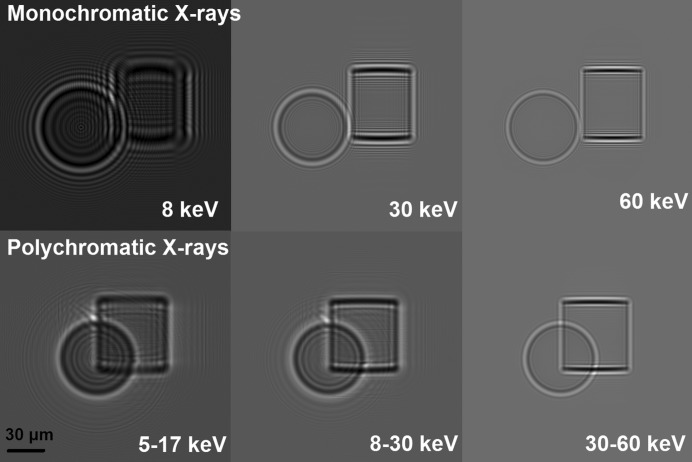
Radiographic simulations of phase-contrast imaging of PMMA samples. A sphere with a radius of 30 µm and a cylinder with a radius of 30 µm and a height of 60 µm were simulated.

Fig. 3 ▶ shows simulated radiographic X-ray images of PMMA samples. They are a sphere and a cylinder with the same diameter of 60 µm. The top row of Fig. 3 ▶ shows results using monochromatic X-ray radiation at the following energies: 8, 30, 60 keV. The results of imaging in the case of polychromatic X-rays are depicted in the bottom row of Fig. 3 ▶. The visibility of interference fringes increases with decreasing energy. It is possible to observe the same phenomenon using both polychromatic and monochromatic radiation. This confirms that the visibility of phase contrast for this kind of material is better when using lower energies. The visibility of interference fringes and their spatial separation from one another is important for the phase-retrieval method, which is used below in §4.1.

## 4. Processing of phase-contrast data

**Figure 4:**
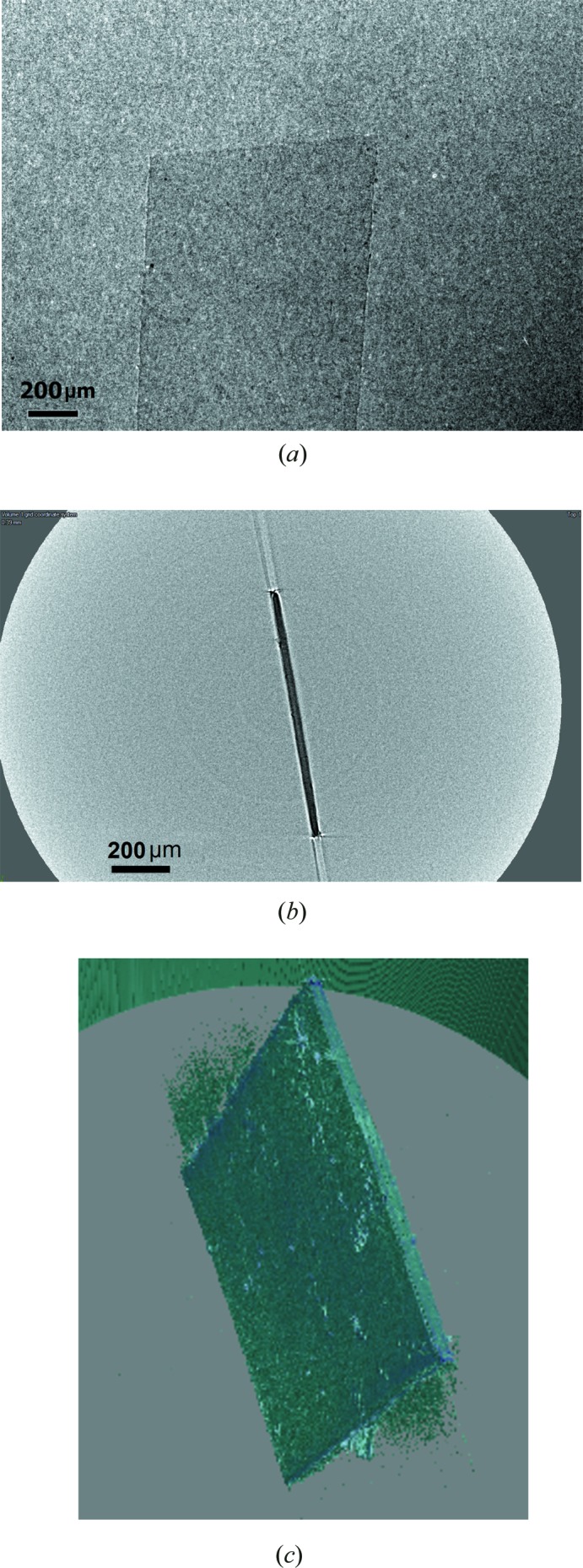
(*a*) Single projection of 30 µm-thick Kapton foil showing very poor contrast. SDD = 109.3 mm, SOD = 28 mm, effective pixel size = 1.6 µm. Total number of projections 2250. Exposure time 1.5 s, setting of X-ray tube 80 kV, 100 µA, focus size of 8 µm. (*b*) Slice from a reconstruction performed using the *Octopus* package. (*c*) Segmentation and visualization using *VGStudio* is complicated owing to ray aliasing artefacts and the remains are visible at the edges of the object.

Fig. 4 ▶ illustrates the results of CT obtained using a free-space-propagation X-ray imaging technique. The results are compatible with the simulation in Fig. 2 ▶, with one difference, that the CT slice in Fig. 4 ▶(*b*) contains more ray aliasing artefacts (Barret & Keat, 2004 ▶) and its remains are visible at the edges of the reconstructed object in Fig. 4 ▶(*c*). This kind of artefact may be intensified by stronger phase-contrast expression along the longer edges of the sample.

**Figure 5:**
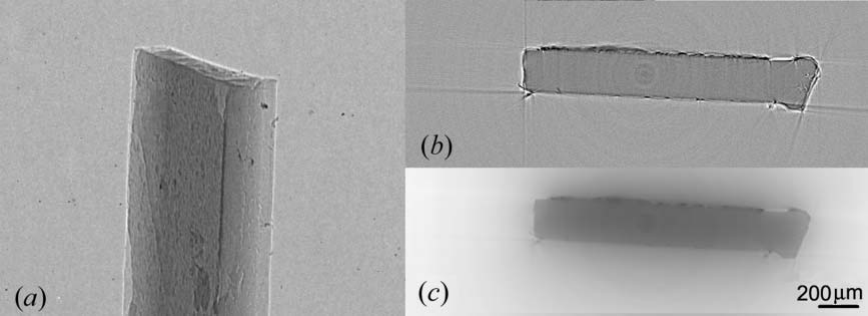
(*a*) X-ray projection of a plastic foil, (*b*) reconstructed CT slice using *Octopus* and (*c*) reconstructed CT slice using *X-TRACT*.

One of thousands of X-ray projections of a plastic foil with a 3 µm layer of paint on one side with the phase contrast resolved on the edges is depicted in Fig. 5 ▶(*a*). The setting of the geometry for the experiment results in a magnification factor of 3.75 at full power of the X-ray tube, 80 kV and 100 µA. Fig. 5 ▶(*b*) shows a reconstructed CT slice using a standard reconstruction algorithm in *Octopus*. Dark and white stripes appear close to the three-dimensional reconstructed structure, known as ray aliasing. Fig. 5 ▶(*c*) shows a reconstructed CT slice using the *X-TRACT* software. It shows a suppression of ray artefacts, but it also gives slightly dark hazing near the object, which can cause problems for voxel segmentation.

### 4.1. Phase-retrieval method

The phase-retrieval algorithm in the *ANKAphase* application (Weitkamp *et al.*, 2011 ▶) was used for phase retrieval in the Kapton foil. The program uses the single-distance non-iterative phase-retrieval algorithm described by Paganin *et al.* (2002 ▶). It was applied to the projections described in Fig. 4 ▶(*a*). The intensity distribution *I*(*x*, *y*) in Fig. 4 ▶(*a*) measured at a single known distance *Z* between the object and the detector plane can be used to retrieve the projected thickness *t*(*x*, *y*) of the object and thus we get a better contrast in the image, which is equivalent to the projected phase shift of the X-ray wavefront.

**Figure 6:**
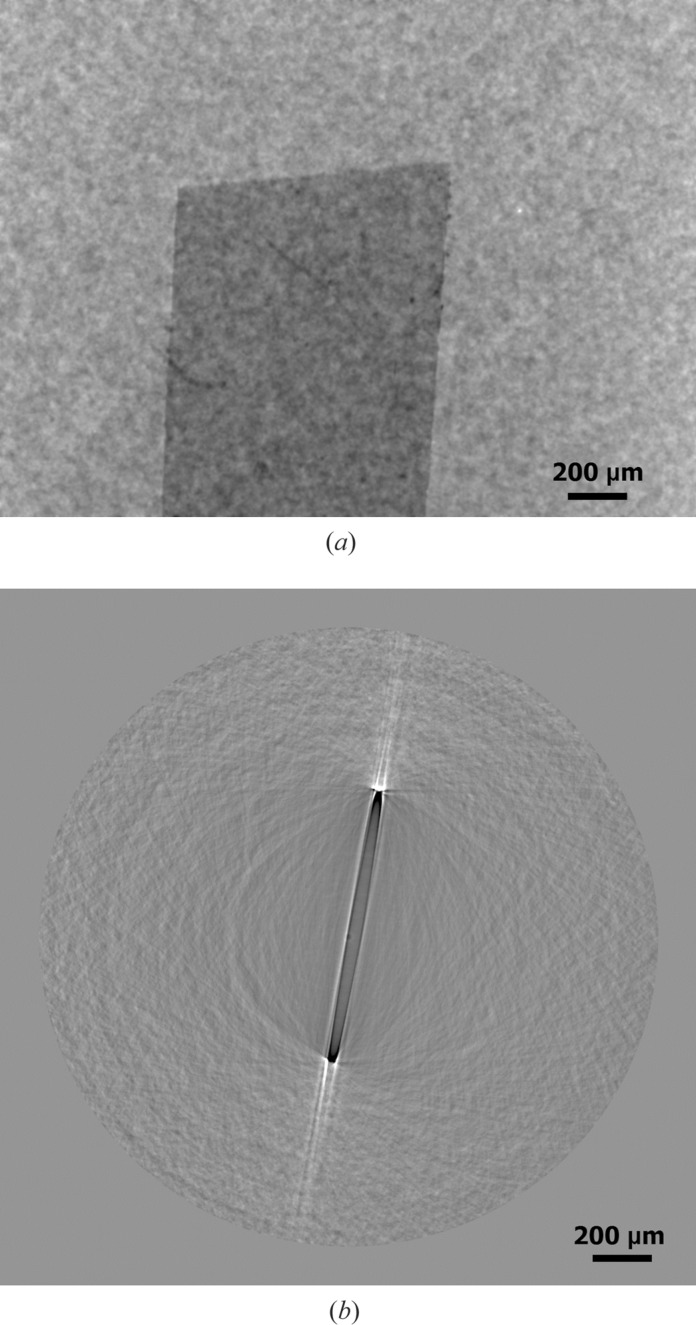
(*a*) Phase map resulting from *ANKAphase* application and (*b*) CT slice as a result of CT reconstruction of phase map projections.

The algorithm is strictly valid only if the following experimental conditions are fulfilled. The object imaged consists of a single, homogeneous material. Monochromatic radiation is used. The distance *Z* between the object and the detector plane fulfils the near-field condition. Although not all conditions of the algorithm have been fulfilled (polychromatic radiation was used), we can see a significant improvement in the visibility of the phase object. On the other hand, there was a significant deterioration in the CT reconstruction (see Fig. 6 ▶ *b*). It was difficult to obtain a three-dimensional image by voxel segmentation.

**Figure 7:**
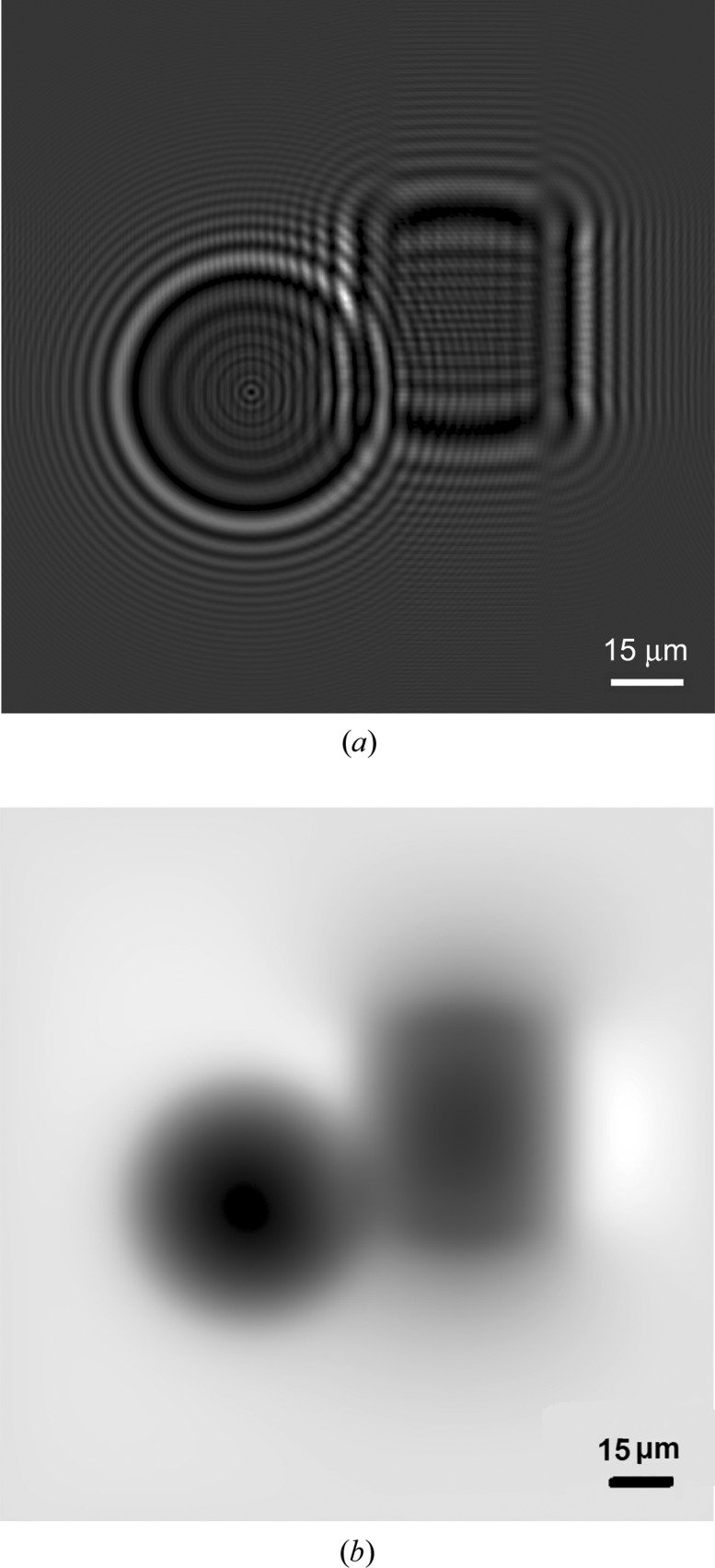
(*a*) Simulation of a single X-ray projection of the PMMA samples (sphere, cylinder) and (*b*) distribution of reconstructed phase map in the object plane after using phase-retrieval algorithm TIE1.

The *X-TRACT* application was used for the purpose of phase retrieval in the case of the PMMA samples. The TIE1 algorithm recovers the optical phase of an electromagnetic wave from a single near-field image by solving the transport of intensity equation. In the case of a polychromatic incident wave, the result allows us to see more clearly the transverse variations of the projected density of the sample. First, we used this algorithm for the simulated objects depicted in Fig. 7 ▶(*a*) for an X-ray energy of 8 keV. For the algorithm, it is necessary to specify input data concerning the sample material and the X-ray energy used. The chemical formula of PMMA is C_5_H_8_O_2_, and the density is 1.19 g cm^−3^. Index of refraction decrements δ and β must be entered. The *X-TRACT* application requires the ratio of δ and β. For an X-ray energy of 8 keV, δ = 4.2 × 10^−6^ and β = 9.2 × 10^−9^. The ratio δ/β = 453 (Henke *et al.*, 1993 ▶). The result of the phase-retrieval algorithm TIE1 is shown in Fig. 7 ▶(*b*).

**Figure 8:**
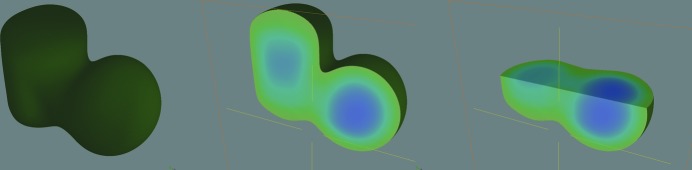
Three-dimensional visualization of a reconstructed three-dimensional phase map of the PMMA samples. An increase of the relative electron density towards the inside of the samples is seen.

The retrieved phase is directly proportional to the projected electron density in the sample and can be used for densitometry and CT. Simulation of 2250 projections was carried out. The plane wave source producing the mono-energetic X-ray beam of 8 keV was used. The source to sample distance (SSD) was 2 m and the detector centre to sample origin distance (SDD) was 0.5 m. Phase maps were retrieved from these projections. These maps were reconstructed using a standard reconstruction algorithm to obtain CT slices. The three-dimensional phase map in Fig. 8 ▶ has been created by stacking of CT slices and by voxel segmentation. The transition from green to blue shows an increase in the relative electron density towards the inside of the samples.

**Figure 9:**
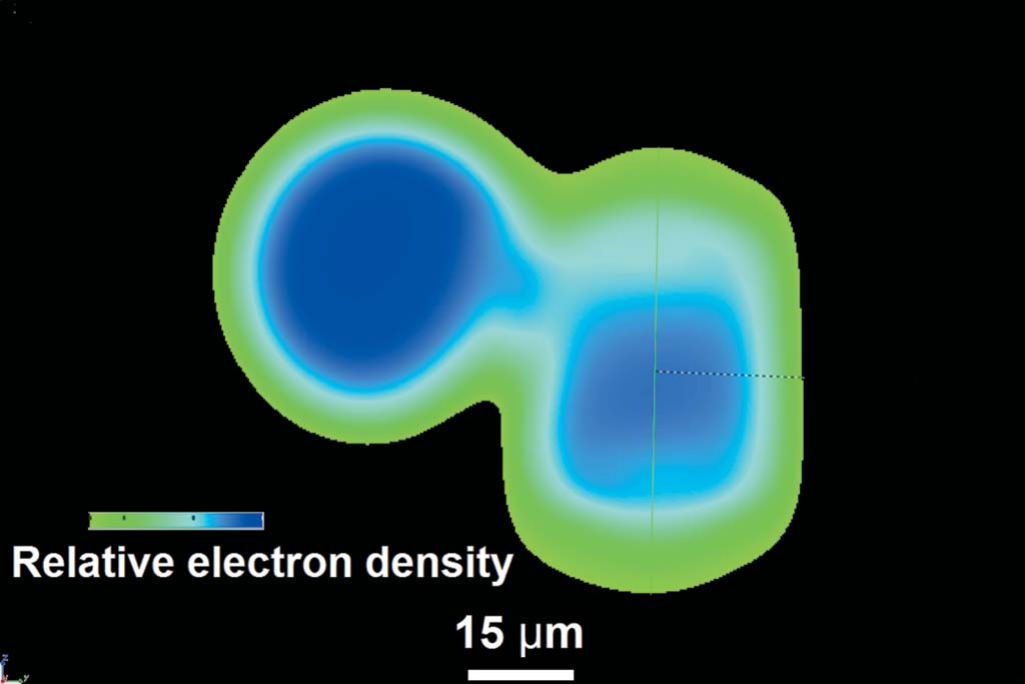
The cross section through the three-dimensional phase map, showing the electron density, which grows towards the inside of the samples.

It is possible to compare the dimensions of the phase map with real input data in the simulation of phase-contrast images using measurement features available for distances in *VGStudio* quite easily. The height of the cylinder of 65 µm is greater (8%) than was specified (60 µm) for the simulated projection. The diameter of the phase distribution (51 µm) for the sphere sample is nearly 10 µm less (15%) than was specified in the simulation. The measurement of distances was done in the slice depicted in Fig. 9 ▶.

**Figure 10:**
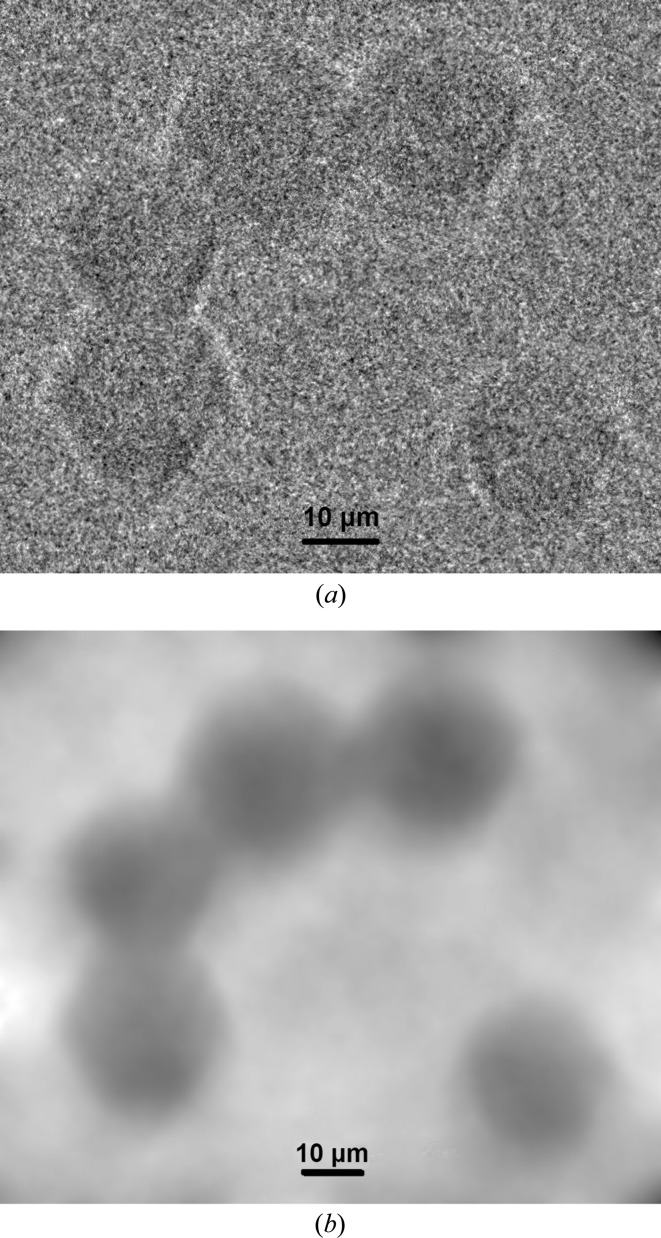
(*a*) Single X-ray projection of PMMA spheres (diameter of 20 µm) after flat-field correction and (*b*) the retrieved phase image (relative electron density) of PMMA spheres.

The single X-ray projection shown in Fig. 10 ▶(*a*) of PMMA spheres (20 µm diameter) deposited on an Si_3_N_4_ (500 nm) membrane was taken in an experiment with a real sample. The exposure time of the X-ray projection and of the flat-field image was 40 s. SSD = 16 mm and SDD = 1043.8 mm, which yields a geometrical magnification of *M* = SDD/SSD = 65. It is possible to calculate the geometrical blur due to finite source size defined by *U*_F_ = *D*_F_(*M* − 1), where *D*_F_ is the focal spot size and *M* is the geometrical magnification (Salamon *et al.*, 2008 ▶). In this case, the blur aberration is equal to 512 µm. However, this value does not have a significant influence on the visibility of samples that have a diameter of the order of 20 µm (see Fig. 10 ▶ *a*). It can be concluded that the divergence of X-ray beams propagating behind the sample is smaller than the calculated geometrical blur aberration. Also the intensity of the blur aberration seems to be low. Fig. 10 ▶(*b*) shows a phase-retrieved image using the TIE1 algorithm in the *X-TRACT* application. Significant increases in the contrast of spheres and the transverse variations of the projected density are observed.

## 5. Conclusion

This contribution shows the results of processing of simulated and real phase-contrast images using a phase-retrieval algorithm in laboratory conditions with a microfocus X-ray source and a high-resolution CCD camera. Phase-contrast objects modify not the amplitude of the X-ray radiation but the phase shift. The X-ray detector is sensitive to the intensity (square of amplitude), so it is not possible to see these variations in phase shifts directly. The *ANKAphase* application was tested first because it is available as a free plug-in in the *ImageJ* software (Rasband, 2012 ▶). For now, it has been tested only for one type of sample (Kapton foil) and comparisons with other available algorithms will be made in future work. In the case of the PMMA samples, phase-retrieved images were obtained using the TIE1 algorithm in the *X-TRACT* application. One of the strict conditions is using a homogeneous sample. This condition was fulfilled in simulated as well as in real experiments. The three-dimensional phase map has been created only in the case of simulation. Tomographic reconstruction of the retrieved phase images gave us information about the relative electron density in the object. Comparison of the object dimensions in the phase map with input object data in the simulation of X-ray projections shows differences in the range of 8–15%. The experiment with the real sample (PMMA spheres) shows successful application of the phase-retrieval algorithm for a single radiographic X-ray projection. The phase image obtained is very compatible with the simulated image. The phase image visibility of real objects, similarly to simulated objects, was increased. Tomographic reconstruction was not carried out because of the small size of the samples, which is a challenge for our future work.
